# Comparison of Binax NOW urine antigen test and pneumococcal DNA assay using qPCR before and after nasopharyngeal swabbing in healthy Malawian children

**DOI:** 10.1016/j.nmni.2015.08.001

**Published:** 2015-08-14

**Authors:** E.A. Lees, D.K.K. Ho, M. Guiver, L.A. Mankhambo, N. French, ED Carrol

**Affiliations:** 1) Department of Clinical Pharmacology, University of Liverpool Institute of Translational Medicine, United Kingdom; 2) Department of Women's and Children's Health, University of Liverpool Institute of Translational Medicine, Alder Hey Children's NHS Foundation Trust, United Kingdom; 3) Department of Clinical Infection, Microbiology and Immunology, University of Liverpool Institute of Infection and Global Health, Liverpool, United Kingdom; 4) Public Health England North West, Manchester Laboratory, Manchester Medical Microbiology Partnership, Manchester Royal Infirmary, Manchester, United Kingdom; 5) Malawi–Liverpool–Wellcome Trust Clinical Research Programme, Malawi

**Keywords:** Binax NOW urine antigen test, DNAaemia, invasive pneumococcal disease, nasopharyngeal swabbing, pneumococcal PCR

## Abstract

Diagnosis of invasive pneumococcal disease is challenging. We compared Binax NOW pneumococcal urinary antigen test with blood pneumococcal PCR in healthy Malawian children with and without pneumococcal carriage, and we found a high false-positive rate with Binax NOW. Blood pneumococcal PCR positivity was 66/88 (75%) compared to 5/27 (18%) when nasopharyngeal swabbing was performed first compared to after blood sampling for pneumococcal blood PCR. We speculate that nasopharyngeal swabbing may be causing a breach of mucosal integrity, leading to invasion into the bloodstream. These findings need to be confirmed with autolysin-based PCR assays.

## Introduction

Invasive pneumococcal disease (IPD) is a major contributor to mortality in the developing world, especially in the context of HIV [1]. The diagnosis of IPD is problematic, particularly for pneumococcal pneumonia, because of a low rate of culture-positive patients, suggesting that the true disease burden is likely to be underestimated. Equally, the distinction between nasopharyngeal carriage and IPD is an important one for children in sub-Saharan Africa in order to reliably quantify disease burden.

In children with pneumonia, blood pneumococcal PCR (based on pneumolysin) was positive in 100% of children with positive blood cultures and in only 5% of healthy controls [2]. The Binax NOW (Binax, Portland, ME, USA) *Streptococcus pneumoniae* antigen test is a rapid assay for the qualitative detection of *S. pneumoniae* antigen in the urine of patients with pneumonia and detects C-polysaccharide antigen. In children with pneumonia, Binax NOW has been shown to have a sensitivity and specificity of 87% and 63%, respectively, for diagnosis of pneumococcal pneumonia, but pneumococcal antigen was also detected in 43% of urine samples from healthy nasopharyngeal *S. pneumoniae* carriers [3]. Binax NOW has been associated with false-positive findings, especially in children with high rates of nasopharyngeal pneumococcal colonization [4–6].

## Patients and methods

We aimed to compare the performance of Binax NOW urine antigen test and a real-time blood PCR assay in healthy Malawian children with pneumococcal nasopharyngeal carriage before and after nasopharyngeal swabbing (NPS). The study was conducted at Queen Elizabeth Central Hospital in Blantyre, Malawi. Between April 2004 and April 2006, children who presented with meningitis and pneumonia were recruited as cases into a study examining genetic susceptibility to IPD, and healthy asymptomatic children from the same villages served as controls [7]. A control was a healthy, afebrile, malaria aparasitaemic child from the same village as the case. For each index case, at least three healthy, age-matched children were selected from the neighborhood. Pneumococcal conjugate vaccine had not yet been introduced into Malawi when the study was conducted. NPS, urine sample for Binax NOW antigen testing and a finger-prick blood sample for pneumococcal PCR and malaria parasites were collected. NPS samples were collected in STGG medium and cultured within 4 hours on sheep's blood and chocolate agar, and presumptive *S. pneumoniae* colonies were identified on the basis of their morphology and Optochin sensitivity. Pneumococcal carriage was defined as growth of *S. pneumoniae* on culture from a NPS. Binax NOW urine antigen testing was performed according to the manufacturer's instructions. Pneumococcal bacterial DNA (pneumolysin) was amplified and quantified using real-time PCR as previously described [8,9]. Briefly, this is a single-tube multiplex PCR assay which was developed based on *N. meningitidis, S. pneumoniae* and *H. influenzae* targets. The pneumococcal PCR component is specific for 23 pneumococcal serotypes while simultaneously offering a high level of sensitivity. In this study, a cutoff value of 100 copies/mL was used *a priori* to define a positive pneumococcal PCR test. This cutoff was chosen on the basis of previous literature. The plasma *ply* PCR used by Abdeldaim *et al*. [10] reported that all positive samples had copy numbers >10^3^/mL. The study was approved by the University of Malawi College of Medicine research ethics committee.

## Results

We recruited 118 healthy, asymptomatic children, of whom 8/118 (6.8%) were HIV infected. There were 63 boys (53.4%) with a median age of 5.9 years (interquartile range (IQR), 3.8–8.3 years). Pneumococcal carriage rate was 42.4% (50/118). Overall, Binax NOW was positive in 22/51 (44%) children with pneumococcal carriage and 21/68 (30.9%) without carriage (p > 0.05) (Table 1). In the first 88 children, NPS was performed before blood sampling as a result of concerns that the children might become upset after blood sampling and refuse NPS. When the swabs were done before the blood sampling was performed for pneumococcal PCR, 66/88 (75%) children had a positive pneumococcal blood PCR, with a median pneumococcal bacterial load of 2040 copies/mL (range, 113–511 518 copies/mL; IQR, 464–15842 copies/mL). The Bacterial load was higher in children in whom NPS was performed before blood sampling, but this difference was no longer significant after adjusting for age and HIV status (Table 2). When swabbing was performed first, 22/50 (44%) children with pneumococcal carriage had a positive Binax NOW test compared with 13/38 (34%) without pneumococcal carriage (p > 0.05).

Following these results, we speculated that NPS was inducing a transient pneumococcal DNAaemia, so we performed blood sampling for pneumococcal PCR before NPS in the remaining children. When blood sampling was performed first, 5/27 (18%) had a positive pneumococcal PCR, with a median bacteria load of 2369 copies/mL (range, 1996–263 597 copies/mL; IQR, 2101–134 114 copies/mL). When blood tests were taken first, 8/30 (27%) without pneumococcal carriage had a positive Binax NOW result. (None of these children had pneumococcal carriage.)

## Discussion

Our data are consistent with previous studies suggesting that Binax NOW has a high false-positive rate in asymptomatic children colonized with *S. pneumoniae*. The high false-positive rate even in those without pneumococcal carriage suggests that our carriage detection rate was an underestimate.

Interestingly, our results suggest that NPS can induce a significant pneumococcal DNAaemia; this warrants further study. Assessment of pneumococcal bacterial load should be performed before NPS to avoid artifactual increases in bacterial load; however, pneumococcal DNAaemia also occurred in healthy colonized children before NPS. We speculate that the process of NPS may be creating some sort of friction on the mucosal membrane, altering the integrity of the epithelial barrier and allowing invasion into the bloodstream.

PCR-based assays for identifying *S. pneumoniae* targeting pneumolysin (*ply*) should ideally, be specific for *S. pneumoniae.* However, viridans group streptococci (*S. mitis* or *S. oralis*) have been reported to occasionally harbour genes encoding *S. pneumoniae* virulence factors [11]. Nonetheless, in our study, when the same assay was performed before NPS, the positivity rate was significantly lower. Pneumolysin PCR has been reported to be not specific enough for routine use with small plasma volumes for the detection of nonbacteraemic pneumococcal pneumonia; however, our assay uses whole blood, not plasma. We previously showed that the sensitivity of a meningococcal PCR for confirming clinical meningococcal disease improved significantly with a new whole-blood TaqMan PCR compared to a serum TaqMan PCR method. Lack of specificity is also seen with the pneumococcal *lyt*A PCR assay. Although current practice in our laboratory is to routinely run both *ply* and *lyt*A assays for confirmation of positive pneumococcal PCR, at the time of our study, only *ply* was used. Our data highlight the challenges and limitations of using *ply*-based PCR assays for the diagnosis of IPD in resource-poor settings where there is a high prevalence of pneumococcal carriage in children. Larger studies are required to assess the performance of different pneumococcal PCR assays in healthy controls with and without pneumococcal carriage.

## Conflict of interest

None declared.

## Figures and Tables

**Table 1:** Binax NOW and pneumococcal PCR positivity in children with and without pneumococcal carriage

Test	No carriage	Carriage	p
Binax NOW	21/68 (30.9%)	22/50 (44%)	NS
Pneumococcal PCR	28/65 (43.1%)	43/50 (82%)	<0.0005
Total	68	50	

Pneumococcal PCR was not performed in three patients.

**Table 2:** Binax NOW and pneumococcal PCR positivity in children before and after nasopharyngeal swabbing

Test	Pneumococcal PCR after swabbing	Pneumococcal PCR before swabbing	p
Binax NOW positive	35/88 (39.8%)	8/30 (26.7%)	>0.05
Pneumococcal PCR positive	66/88 (75%)	5/27 (18.5%)	<0.0005
Total	88	30	

Pneumococcal PCR was not performed in three patients.
